# A review of the direct and indirect effects of neonicotinoids and fipronil on vertebrate wildlife

**DOI:** 10.1007/s11356-014-3180-5

**Published:** 2014-06-18

**Authors:** David Gibbons, Christy Morrissey, Pierre Mineau

**Affiliations:** 1 RSPB Centre for Conservation Science, RSPB, The Lodge, Sandy, Bedfordshire, SG19 2DL UK; 2 Department of Biology, University of Saskatchewan, 112 Science Place, Saskatoon, Saskatchewan S7N 5E2 Canada; 3 School of Environment and Sustainability, University of Saskatchewan, 117 Science Place, Saskatoon, Saskatchewan S7N 5E2 Canada; 4 Pierre Mineau Consulting, 124 Creekside Drive, Salt Spring Island, V8K 2E4 Canada

**Keywords:** Pesticide, Neonicotinoid, Imidacloprid, Clothianidin, Fipronil, Vertebrate, Wildlife, Mammals, Birds, Fish, Amphibians, Reptiles, Risk assessment

## Abstract

Concerns over the role of pesticides affecting vertebrate wildlife populations have recently focussed on systemic products which exert broad-spectrum toxicity. Given that the neonicotinoids have become the fastest-growing class of insecticides globally, we review here 150 studies of their direct (toxic) and indirect (e.g. food chain) effects on vertebrate wildlife—mammals, birds, fish, amphibians and reptiles. We focus on two neonicotinoids, imidacloprid and clothianidin, and a third insecticide, fipronil, which also acts in the same systemic manner. Imidacloprid and fipronil were found to be toxic to many birds and most fish, respectively. All three insecticides exert sub-lethal effects, ranging from genotoxic and cytotoxic effects, and impaired immune function, to reduced growth and reproductive success, often at concentrations well below those associated with mortality. Use of imidacloprid and clothianidin as seed treatments on some crops poses risks to small birds, and ingestion of even a few treated seeds could cause mortality or reproductive impairment to sensitive bird species. In contrast, environmental concentrations of imidacloprid and clothianidin appear to be at levels below those which will cause mortality to freshwater vertebrates, although sub-lethal effects may occur. Some recorded environmental concentrations of fipronil, however, may be sufficiently high to harm fish. Indirect effects are rarely considered in risk assessment processes and there is a paucity of data, despite the potential to exert population-level effects. Our research revealed two field case studies of indirect effects. In one, reductions in invertebrate prey from both imidacloprid and fipronil uses led to impaired growth in a fish species, and in another, reductions in populations in two lizard species were linked to effects of fipronil on termite prey. Evidence presented here suggests that the systemic insecticides, neonicotinoids and fipronil, are capable of exerting direct and indirect effects on terrestrial and aquatic vertebrate wildlife, thus warranting further review of their environmental safety.

## Overview of impacts of pesticides on vertebrate wildlife

Although vertebrates are the intended target of only 2 % of pesticides on the market, the unintentional impacts of pesticides on vertebrate populations have been marked and are well documented (e.g. Sánchez-Bayo 2011). Pesticides can exert their impact on vertebrates either directly, through their toxicity, or indirectly, for example, by reducing their food supply.

*Direct effects* may be the result of several different exposure pathways: through ingestion of the formulated product (e.g. birds eating seeds coated with insecticide; Avery et al. 1997; Prosser and Hart 2005), through uptake via the skin following a spray event (Mineau 2011) or by eating contaminated prey. Probably the most notable example among the latter exposure pathway was the dramatic impact that organochlorine pesticides, especially DDT and its metabolite DDE, had on populations of birds of prey (Ratcliffe 1967; Newton 1995). Depending on the extent of intoxication, direct effects of pesticides can either kill vertebrates outright or exert sub-lethal effects, for example, on growth and reproduction (Sánchez-Bayo 2011). Progress since the organo-chlorine era has helped ensure that compounds that are currently being developed and registered are generally less persistent and do not as readily bio-accumulate in food webs.

More recently, however, interest has turned to investigating the potential for *indirect effects* which are typically mediated through loss in quantity or quality of prey associated with pesticide use, or through habitat modification (Sotherton and Holland 2002; Boatman et al. 2004; Morris et al. 2005). This is especially the case in jurisdictions where the use of highly toxic pesticides has been controlled and the frequency of direct impacts reduced (Mineau et al. 1999).

Over the last 2 decades, a new class of insecticides, the neonicotinoids, has become the most important and fastest growing of the five major chemical classes of insecticides on the global market (Jeschke and Nauen 2008; Jeschke et al. 2011; Tomizawa and Casida 2011; Casida and Durkin 2013). When used as plant protection products, neonicotinoids act by becoming distributed systemically throughout the growing plant following seed or soil applications. Another recent insecticide, fipronil, a phenyl-pyrazole (fiprole) rather than a neonicotinoid, also acts in the same manner and has a similar toxicity and persistence profile (Grant et al. 1998). Consequently, the neonicotinoids and fipronil are sometimes jointly termed ‘systemic insecticides’, although there are also older products which could be termed ‘systemic’, for example, the organo-phosphorous insecticide acephate and the organo-arsenical, monosodium methanearsonate. Neonicotinoids are, in particular, commonly applied as seed treatments. The use of seed treatments as a convenient and effective application method has widespread appeal in the farming industry. Consequently, systemic seed treatments are now used on the majority of agricultural crops worldwide (Garthwaite et al. 2003; Jeschke et al. 2011).

Here, we build on the reviews of others (e.g. Goulson 2013; Köhler and Triebskorn 2013; Mineau and Palmer 2013) to examine the evidence and potential for direct and indirect effects of two common systemic neonicotinoid insecticides, imidacloprid and clothianidin, along with fipronil on vertebrate wildlife.

## Mode of action of the systemic insecticides

Neonicotinoids work by interfering with neural transmission in the central nervous system. They bind to the nicotinic acetylcholine receptors (*n*AChR) in the postsynaptic neuron, acting as ‘false neurotransmitters’ (agonists). This interference with acetylcholine neurotransmitter signalling causes continuous activation of the receptor, leading to symptoms of neurotoxicity. Neonicotinoids have greater affinity for, and thus bind more strongly to, insect than mammalian or other vertebrate receptors, so their toxicity to mammals is lower than it is to insects and the reversibility of intoxication higher (Tomizawa and Casida 2005; Jeschke et al. 2011). Fipronil works similarly, but instead binds to the gamma-aminobutyric acid (GABA) receptors, resulting in similar continuous central nervous system activity (Tingle et al. 2000, 2003). As with neonicotinoids, fipronil has a lower affinity to vertebrate than to invertebrate receptors (Grant et al. 1998). Despite the lower toxicity of these products to vertebrates than to invertebrates, there is still ample evidence that vertebrates show toxic effects, albeit at markedly higher concentrations than for many target and non-target invertebrate species (e.g. Tingle et al. 2000, 2003; Cox 2001; SERA 2005; DeCant and Barrett 2010; Mineau and Palmer 2013).

## Materials and methods

To assess the likely impacts of neonicotinoids and fipronil on vertebrates, a literature search was undertaken using Web of Science and Google Scholar. Search terms were [product] and [taxon], where [product] was either neonicotinoid, imidacloprid, thiacloprid, clothianidin, thiamethoxam, acetamiprid, nitenpyram, dinotefuran or fipronil; and [taxon] was either vertebrate*, mammal*, bird*, reptile*, amphibian* and fish*. In addition, specific searches were made on a few common toxicity test species (e.g. rat) and by following up references cited in the publications found by the search. The review also draws heavily on the recently published report by Mineau and Palmer (2013) on the direct and indirect toxicity of neonicotinoids to birds. Several industry studies, which have not been formally published but which were part of product approval processes, were reviewed by Mineau and Palmer and have been included here. While industry studies have been reviewed by regulators and may receive as much critical review as in the open peer-reviewed literature, emphasis here is on published reports and the primary literature.

The following information was extracted from each study: the product used, its dose and whether or not it was presented as a single dose (acute) or over a period of time (chronic; e.g. over 30 days); the effects on individual organisms, specifically whether there was an impact on survival, reproduction, growth and development, or other sub-lethal effects, such as neurobehavioural, genotoxic, cytotoxic, and immunotoxic; the impact on populations of the animal (e.g. local populations); the type of study, separated into laboratory or field; and finally whether it was a study of direct toxic effects, or indirect effects (e.g. leading to changes in food availability). In some cases, individual studies covered more than one species, and each is treated here as a separate species impact study.

The great majority of the studies were laboratory-based (139/152 = 91 %) and most (146, 96 %) were direct toxicity studies. While common in ecotoxicology, the lack of field testing and over-reliance on laboratory direct toxicity testing limit our ability to interpret the findings under field-realistic conditions. Field experiments have provided some of the most compelling evidence of the impact of neonicotinoids on populations in their natural environment (e.g. Whitehorn et al. 2012), and there is an increasing recognition that maintaining ecological complexity in field studies is desirable (Suryanarayanan 2013).

The most common study taxa were mammals (58), birds (47) and fish (32), with substantially fewer studies of amphibians (12) and reptiles (3). Within these individual taxa, the most commonly studied mammals were rat, *Rattus norvegicus*, (39) and mouse, *Mus musculus*, (9); the most commonly studied birds were northern bobwhite quail, *Colinus virginianus*, (8) and mallard, *Anas platyrhynchos*, (6), the two test species mandated by regulatory approval schemes in North America; and the most commonly tested fish were rainbow trout, *Oncorhynchus mykiss*, (6) and Nile tilapia, *Oreochromis niloticus*, (6).

Most of these studies investigated the effects of the two neonicotinoids, imidacloprid (72) and clothianidin (19), as well as fipronil (47); between them, these three insecticides accounted for 91 % of all studies. Given the paucity of information collated for the other neonicotinoids, this review concentrates on these three products alone.

## The direct effects of neonicotinoids and fipronil on vertebrate wildlife

### Toxicity to vertebrates

Standard toxicity testing for pesticides on terrestrial vertebrates is through an acute (<96 h) study. Test organisms are given the product by gavage (i.e. through a feeding tube) or through the diet in varying concentrations, and the estimated dose of pesticide associated with death of half of the test subjects is recorded and expressed as a proportion of bodyweight (i.e. the 50 % lethal dose, LD_50_, expressed as milligrams of pesticide per kilogram of bodyweight). Toxicity for aquatic organisms is typically measured as the LC_50_ or the concentration in water (e.g. mg/L) which is toxic to the test organisms. Numerous LD_50_ and LC_50_ tests have been undertaken for vertebrates, and those that were located as part of this review are shown for imidacloprid, clothianidin and fipronil in Table 1. As can be seen, the relative toxicity of these products varies, both among products and among species.

**Table 1:** Single (acute) dose LD_50_ (for mammals birds and reptiles, mg/kg) and LC_50_ (for fish and amphibia, mg/L) for imidacloprid, clothianidin and fipronil

Taxon	Species	Imidacloprid	Clothianidin	Fipronil
Mammal	Rat, *Rattus norvegicus*	425-475 (MT)^a^	5,000 (PNT)^i^	97 (MT)^l^
	Mouse, *Mus musculus*	131-300 (MT)^a^	>389 (MT)^i^	95 (MT)^m^
Bird	Mallard, *Anas platyrhynchos*	283 (MT)^b^	>752 (ST)^j^	2,150 (PNT)^l^
	Ring-necked pheasant, *Phasianus colchicus*			31 (HT)^l^
	Grey partridge, *Perdix perdix*	13.9 (HT)^c^		
	Red-legged partridge, *Alectoris rufa*			34 (HT)^l^
	Northern bobwhite quail, *Colinus virginianus*	152 (MT)^a^	>2,000 (PNT)^k^	11.3 (HT)^l^
	Japanese quail, *Coturnix japonica*	31 (HT)^a^	423 (MT)^k^	
	Feral pigeon, *Columba livia*	25–50 (HT)^a^		>2,000 (PNT)^l^
	House sparrow, *Passer domesticus*	41 (HT)^a^		
	Field sparrow, *Spizella pusilla*			1,120 (ST)^l^
	Canary, *Serinus canaria*	25–50 (HT)^a^		
	Zebra finch, *Taeniopygia guttata*			310 (MT)^n^
Fish	Bluegill sunfish, *Lepomis macrochirus*	105 (PNT)^a^	>117 (PNT)^i^	0.083 (VHT)^l^
	Japanese carp, *Cyprinus carpio*			0.34 (HT)^l^
	Nile tilapia, *Oreochromis niloticus*			0.042-0.147 (VHT-HT)^l^
	Rainbow trout, *Oncorhynchus mykiss*	>83–211 (ST-PNT)^a^	>105 (PNT)^i^	0.246 (HT)^l^
	Rainbow trout (fry)	1.2 (MT)^d^		
	Sheepshead minnow, *Cyprinodon variegatus*	161 (PNT)^a^	>93.6 (ST)^i^	0.13 (HT)^l^
	Zebrafish, *Danio rerio*	241 (PNT)^e^		
Amphibia	Black-spotted pond frog, *Rana nigromaculata*	129–219 (PNT)^a,f^		
	Indian rice frog, *Rana limnocharis*	82–366 (ST-PNT)^a,f,g^		
	Western chorus frog, *Pseudacris triseriata*	194 (PNT)^h^		
	American toad, *Bufo americanus*	234 (PNT)^h^		
Reptile	Fringe-toed lizard, *Acanthodactylus dumerili*			30 (HT)^o^

Toxicity classification follows US EPA (2012): *PNT* practically non-toxic, *ST* slightly toxic, *MT* moderately toxic, *HT* highly toxic, *VHT* very highly toxic. For birds, mammals and reptiles: PNT >2,000, ST 501–2,000, MT 51–500, HT 10–50, VHT <10. For aquatic organisms, fish and amphibia: PNT >100, ST >10-100, MT >1-10, HT 0.1-1, VHT <0.1. Note that kg in the LD_50_ units refers to body weight of the dosed animal. Source references denoted by superscripts are as follows: ^a^SERA 2005, ^b^Fossen 2006, ^c^Grolleau 1991 in Anon 2012, ^d^Cox 2001, ^e^Tisler et al. 2009, ^f^Feng et al. 2004, ^g^Nian 2009, ^h^Howard et al. 2003, ^i^DeCant and Barrett 2010, ^j^European Commission 2005, ^k^Mineau and Palmer 2013, ^l^Tingle et al. 2003, ^m^Connelly 2011, ^n^Kitulagodage et al. 2008 (NB : a formulation of fipronil containing the dispersant solvent diacetone alcohol was sevenfold more toxic than technical grade fipronil itself), ^o^Peveling and Demba 2003 (NB: 42 %, rather than 50 %, mortality)

The US Environmental Protection Agency has developed an ecotoxicity classification based on LD_50_ and LC_50_ assessments (US EPA 2012). They classify the acute toxicity of a given product on a particular species as either practically non-toxic, slightly toxic, moderately toxic, highly toxic, or very highly toxic based on lethality dose ranges. Sub-lethal or reproductive effects are not included in this classification. By US EPA’s definitions, and within the highly restricted range of species assessed, imidacloprid shows moderate to high toxicity to birds, particularly for smaller-bodied species such as house sparrows, *Passer domesticus*, and canaries, *Serinus canaria*, and approaches very high toxicity to grey partridge, *Perdix perdix*. It is moderately toxic to rats and mice, but practically non-toxic to fish (with the exception of rainbow trout, especially their fry) and amphibians. Clothianidin’s toxicity ranges from moderate to practically non-toxic for both birds and mammals, whereas for the fish studied, it varies from slightly toxic to practically non-toxic. By contrast, for all fish species studied, fipronil is either highly or very highly toxic (e.g. bluegill sunfish, *Lepomis macrochirus*). Fipronil is in addition highly toxic to the three game birds studied (red-legged partridge, *Alectoris rufa*, ring-necked pheasant, *Phasianus colchicus*, and northern bobwhite quail), and moderately toxic to mice and rats.

One of the serious failings of current risk assessments is the underestimation of interspecies variation in insecticide susceptibility that is apparent from Table 1. Too few species are typically tested to derive the true variation in response from the vast array of exposed species in the wild. Mineau and Palmer (2013) discuss this at length for neonicotinoids and propose improved thresholds derived from species sensitivity distributions and estimated ‘hazard doses’ (HD_5_—the LD_50_ value for a species at the 5 % tail of the sensitivity distribution).

### Impacts on growth, development and reproduction of vertebrates

While not necessarily causing mortality among adults, intoxication by imidacloprid, clothianidin and fipronil can reduce the growth, development and reproduction of individual vertebrates (Table 2). Reproductive effects are manifest in a variety of ways among mammals, but especially as reduced sperm production, adverse effects on the fertilization process, reduced rates of pregnancy, higher rates of embryo death, stillbirth and premature birth, and reduced weights of offspring. Among birds, testicular anomalies and reduced fertilization success, reduced eggshell thickness and embryo size, reduced hatching success and chick survival, and chick developmental abnormalities have all been reported. Weight loss, or impaired weight gain, sometimes associated with reduction or cessation of feeding, occurred within all taxa studied.

**Table 2:** Other studies of the direct effects of imidacloprid, clothianidin and fipronil on vertebrates

Taxon and species	Effect on:	Imidacloprid	Clothianidin	Fipronil	Source and detailed effect
Mammal					
Rat, *Rattus norvegicus*	Reproduction	2, 19, 90 mg/kg/day^a,b,c^	24, 31.2–36.8 mg/kg/day^d,e^	280 mg/kg^f^ 26–28 mg/kg/day^g^	^a^Bal et al. 2012; reduced sperm production ^b^Cox 2001; reduced weight offspring ^c^Gawade et al. 2013; abortions, soft tissue abnormalities and skeletal alterations ^d^Bal et al. 2013; no effect on sperm concentration, mobility or morphology, but reduced weight of epididymis and seminal vesicles ^e^DeCant and Barrett 2010; stillbirths and delayed sexual maturation ^f^Ohi et al. 2004; reduced levels of pregnancy ^g^Tingle et al. 2003; range of effects including reduced fertility and decreased litter size
Rat, *Rattus norvegicus*	Growth and development	10,17,25,100 mg/kg/day^a,b,c,d^	31.2 mg/kg/day^e^ 32 mg/kg^f^	20 mg/kg/day^g^	^a^Cox 2001; reduced weight gain ^b^Cox 2001; thyroid lesions ^c^Bhardwaj et al. 2010; reduced weight and locomotor ability ^d^Cox 2001; atrophy of retina ^e^DeCant and Barrett 2010; reduced weight gain of offspring ^f^Bal et al. 2012; reduced body weight and impact on reproductive organs ^g^Tingle et al. 2003; reduced food consumption and reduced weight gain
Rat, *Rattus norvegicus*	Genotoxic	300 mg/kg^a^	24 mg/kg/day^b^(NE)		^a^Demsia et al. 2007; significant effect on in vitro micronucleus induction in rat erythrocytes ^b^Bal et al. 2013; no effect on sperm DNA fragmentation
Rat, *Rattus norvegicus*	Cytotoxic	<400 mg/kg^a^ 0.21,1,20,45 mg/kg/day^b,c,d,e^			^a^Nellore et al. 2010; blocks to the cholinergic enzyme system ^b^Mohany et al. 2011; oxidative stress and hepatotoxicity, i.e. heavily congested central vein and blood sinusoids in liver ^c^Duzguner and Erdogan 2012; oxidative stress and inflammation caused by altering antioxidant systems ^d^Kapoor et al. 2010; oxidative stress ^e^Toor et al. 2013; hepatotoxicity—dilations of central vein and sinusoids between hepatocytes in liver
Rat, *Rattus norvegicus*	Neurobehavioural	337 mg/kg^a^	>2mM^b^ 18–66 mg/kg/day^c^	<30,140-280^dermal^ mg/kg^d,e^	^a^Abou-Donia 2008; offspring dosed in utero, led to neurobehavioural deficits ^b^de Oliveira et al. 2010; increased release of dopamine ^c^Tanaka 2012; adverse neurobehavioural impacts on pups ^d^Martins 2009; reduced movement ^e^Tercariol and Godinho 2011; increased emotion and fear
Rat, *Rattus norvegicus*	Immunotoxic	0.21, 90 mg/kg/day^a,b^			^a^Mohany et al. 2011; significant effect on leukocyte count, immunoglobulins and phagocytic activity ^b^Gawade et al. 2013; compromised immunity
Mouse, *Mus musculus*	Reproduction	5 mM^a^	18–66 mg/kg/day (NE)^b^		^a^Gu et al. 2013; no impact on sperm mobility, but fertilisation process and zygotes adversely affected ^b^Tanaka 2012; no effect on litter size or weight
Mouse, *Mus musculus*	Growth and development		18–66 mg/kg/day (NE)		Tanaka 2012; no effect on litter size or weight
Mouse, *Mus musculus*	Genotoxic	5 mM (NE)			Gu et al. 2013; no effect on DNA integrity
Mouse, *Mus musculus*	Immunotoxic	10 mg/kg/day			Badgujar et al. 2013; suppressed cell-mediated immune response and prominent histopathological alterations in spleen and liver
Rabbit, *Sylvilagus sp*.	Reproduction	72 mg/kg/day^a^	>25 mg/kg/day^b^		^a^Cox 2001; increased frequency of miscarriage ^b^DeCant and Barrett 2010; increase in premature births
Sheep, *Ovis aries*	Growth and development			0.5 mg/kg/day (NE)	Leghait et al. 2010; no thyroid disruption
Cow, *Bos primigenius*	Cytotoxic	1 mg/kg/day (NE)			Kaur et al. 2006; some modest impacts on plasma biochemistry, but mostly no impact on range of other blood measures
Bird					
Mallard, *Anas platyrhynchos*	Reproduction	16 mg/kg/day	>35 mg/kg/day (NE)		Adapted from figures in Mineau and Palmer (2013)^*^; various effects on reproduction
Chicken, *Gallus gallus domesticus*	Growth and development			37.5 mg/kg	Kitulagodage et al. 2011b; reduced feeding and body mass, and developmental abnormalities of chicks
Chicken, *Gallus gallus domesticus*	Neurobehavioural			37.5 mg/kg	Kitulagodage et al. 2011b; behavioural abnormalities of chicks
Red-legged partridge, *Alectoris rufa*	Survival	31.9-53.4 mg/kg/day			Lopez-Antia et al. 2013; reduced chick survival at low dose, and reduced adult survival at high dose
Red-legged partridge, *Alectoris rufa*	Reproduction	31.9 mg/kg/day			Lopez-Antia et al. 2013; reduced fertilisation rate and chick survival
Red-legged partridge, *Alectoris rufa*	Immunotoxic	53.4 mg/kg/day			Lopez-Antia et al. 2013; reduced immune response
Northern bobwhite quail, *Colinus virginianus*	Reproduction		>52 mg/kg/day		Adapted from figures in Mineau and Palmer (2013)^*^; various effects on reproduction
Northern bobwhite quail, *Colinus virginianus*	Growth and development	24 mg/kg/day^a^		11 mg/kg^b^	^a^Adapted from figures in Mineau and Palmer (2013)^*^; various effects on weight ^b^Kitulagodage et al. 2011a; birds stopped feeding so lost weight
Japanese quail, *Coturnix japonica*	Reproduction	1 mg/kg/day			Tokumoto et al. 2013; testicular anomalies; reductions in embryo length when those males mated with un-dosed females
Japanese quail, *Coturnix japonica*	Genotoxic	1 mg/kg/day			Tokumoto et al. 2013; increased breakage of DNA in males
House sparrow, *Passer domesticus*	Neurobehavioural	6 mg/kg			Cox 2001; in-coordination, inability to fly
Zebra finch, *Taeniopygia guttata*	Reproduction			>1 mg/kg	Kitulagodage et al. 2011b; reduced hatching success
Fish					
Japanese carp, *Cyprinus carpio*	Growth & development			REC (NE)	Clasen et al. 2012; no impact on growth or survival, though biochemical changes
Zebrafish, *Danio rerio*	Reproduction	320 mg/L (NE)			Tisler et al. 2009; no effect on embryos observed
Zebrafish, *Danio rerio*	Growth and development			0.33 mg/L	Stehr et al. 2006; notochord degeneration
Zebrafish, *Danio rerio*	Neurobehavioural			0.33 mg/L	Stehr et al. 2006; locomotor defects in embryos and larvae
Fathead minnow, *Pimephales promelas*	Growth and development		20 mg/L		DeCant and Barrett 2010; reduced weight and length
Fathead minnow, *Pimephales promelas*	Genotoxic			0.03 mg/L	Beggel et al. 2012; changes in gene transcription
Fathead minnow, *Pimephales promelas*	Neurobehavioural			0.14 mg/L	Beggel et al. 2010; impaired swimming; formulation more toxic than technical grade
Nile tilapia, *Oreochromis niloticus*	Growth and development	0.134, <1.34 mg/L^a,b^			^a^Lauan and Ocampo 2013; extensive disintegration of testicular tissue. ^b^Ocampo and Sagun 2007; changes to gonads
Medaka, *Oryzias latipes*	Immunotoxic	0.03–0.24 mg/L (1.5*REC)			Sanchez-Bayo and Goka 2005; juveniles stressed, led to ectoparasite infestation, when concentrations high early in the experiment
Silver catfish, *Rhamdia quelen*	Genotoxic			0.0002 mg/L (NE)	Ghisi et al. 2011; no genotoxic effects
Silver catfish, *Rhamdia quelen*	Cytotoxic			0.0002 mg/L	Ghisi et al. 2011; erythrocyte damage
Amphibia					
Black-spotted pond frog, *Rana nigromaculata*	Genotoxic	0.05 mg/L			Feng et al. 2004; DNA damage at very low concentrations

Acute toxicity studies are given in Table 1 and not repeated here. Dosage could either be acute or chronic, the latter shown as /day (per day). All studies demonstrated deleterious effects at the given dosage, except those marked NE (no effect). Studies marked REC were field-based, with insecticides applied at the manufacturer’s recommended rate; all others are of direct toxicity under laboratory conditions. ‘dermal’ = dermal application. Only studies for which dosage information was readily available are listed. ^*^Lowest feed concentrations causing an effect were transformed to a daily dose assuming an average consumption of 21- and 67-g laboratory feed per day for bobwhite quail and mallard, respectively, and average body weights of 210 and 100 g, respectively

Most of the studies found were required for pesticide registration purposes. In birds, a reproductive test is frequently conducted on standard test species such as the northern bobwhite quail or the mallard. This is a truncated test, which consists of feeding a constant concentration of the pesticide to the study animals and then collecting the eggs and incubating them artificially. There is therefore no inclusion of endpoints to assess the ability of the dosed birds to incubate, hatch or raise their young. The test is a hybrid between single life stage chronic toxicity and a test of true reproductive effects, and has been the subject of analysis and criticism (Mineau et al. 1994, 1996; Mineau 2005). Because of the longer duration of the test, and the occasional pair that fails to bond, spurious variance is introduced, thus decreasing the power to detect reproductive deficits in limited sample sizes. On the other hand, because the birds are offered contaminated diet only, with no other food choice, the test may overestimate realistic exposure in the wild. However, it remains the only test available with which to model non-acute risk in avian wildlife.

### Other sub-lethal impacts on vertebrates

A range of other effects of these insecticides have been documented for vertebrates (Table 2), outside of those reported on survival, growth and development, and reproduction. Among mammals—principally rats and mice—these include genotoxic and cytotoxic effects, neuro-behavioural disorders of offspring (including those dosed in utero), lesions of the thyroid, retinal atrophy, reduced movement, and increased measures of anxiety and fear. House sparrows can become uncoordinated and unable to fly, and studies of Japanese quail and red-legged partridges have reported DNA breakages and a reduced immune response, respectively. Similarly, studies of fish have reported changes in gene transcription, erythrocyte damage, disintegration of gonadal tissue, impaired swimming, notochord degeneration and locomotor defects in embryos and larvae. In one case, medaka fish, *Oryzias latipes*, in experimental rice fields became physiologically stressed (characterized by increased anaerobic metabolism leading to hyperglycemia) following exposure to imidacloprid at 1.5 times the commercially recommended rate of application, and subsequently became susceptible to infestation by the protozoan ectoparasite, *Cychlochaeta* (*Trichodina*) *domerguei* (Sánchez-Bayo and Goka 2005). While the majority of studies documented deleterious impacts from neonicotinoid or fipronil exposure, effective doses have not typically been matched to realistic field exposure conditions.

Many of these, perhaps, more subtle sub-lethal effects (Table 2) occur at much lower concentrations than lethal effects (Table 1). Thus, while single oral doses of 425–475 and 5,000 mg/kg of imidacloprid and clothianidin, respectively, will kill rats, lower daily doses of 0.21–100 and 18–66 mg/kg/day have consistently caused a range of sub-lethal effects. For example, a daily dose of 10–19 or 31 mg/kg/day of imidacloprid and clothianidin, respectively, will cause reduced growth of young rats and, in the case of clothianidin, a greater frequency of stillbirths. Even doses as low as 0.21 and 2.0 mg/kg/day of imidacloprid have been shown to have immunotoxic effects and reduce sperm production, respectively. Similarly, while a single oral dose of 41 mg/kg of imidacloprid will cause mortality in house sparrows, a substantially lower dose (6 mg/kg) can induce uncoordinated behaviour and an inability to fly. While imidacloprid is highly toxic to Japanese quail, with an LD_50_ of 31 mg/kg, chronic daily doses of only 1 mg/kg/day can lead to testicular anomalies, DNA damage in males, and reductions in embryo size when those males are mated with control females. The black-spotted pond frog has an LC_50_ of 129–219 mg/L of imidacloprid, but DNA damage occurs at a much lower concentration, 0.05 mg/L. Given the high toxicity of fipronil to fish, it is perhaps not surprising that the lowest recorded concentration of that insecticide to affect a vertebrate was of 0.0002 mg/L (0.2 μg/L); the effect being erythrocyte damage in silver catfish, *Rhamdia quelen*. While it is difficult to extrapolate such sub-organism effects to fitness-related measures in individuals and population-level responses, they offer insight into potential mechanisms underpinning direct toxicity.

Different families of pesticides rarely elicit sub-lethal effects at doses below 1/10 of the lethal dose (Callahan and Mineau 2008). But, in the case of imidacloprid, signs of severe debilitation (e.g. ataxia) were observed a full order of magnitude below lethal doses. Review of available laboratory data here suggests that some effects can be detected at even lower doses (1/1,000). This apparent feature of these insecticides is of toxicological concern with respect to vertebrates, increasing the probability that wild species can be affected under field-realistic exposure conditions.

### Are vertebrates at risk in their natural environment?

#### Risks to aquatic vertebrates

Various measured or estimated environmental concentrations of imidacloprid, clothianidin and fipronil in the aquatic environment are available. For imidiacloprid, these include 0–0.22 μg/L (Lamers et al. 2011); mean and maximum values of 0.016 and 0.27 μg/L, respectively (Main et al. 2014); 0.13–0.14 μg/L (Stoughton et al. 2008); 0–3.3 μg/L (Starner and Goh 2012); 1–14 μg/L (Jemec et al. 2007); <15 μg/L (Kreuger et al. 2010); 17–36 μg/L (Fossen 2006); and up to 49 μg/L (Hayasaka et al. 2012). Higher concentrations of imidacloprid have been more rarely recorded in the aquatic environment. In one study in the Netherlands, while 98 % of 1,465 measurements ranged from 0 to 8.1 μg/L, the remaining 2 % were up to 320 μg/L (Van Dijk et al. 2013). Similarly, in a study in experimental rice fields, the concentration of imidacloprid immediately after application was 240 μg/L, but fell to 5 μg/L within a week (Sánchez-Bayo and Goka 2005). For clothianidin, DeCant and Barrett (2010) estimated concentrations of 0.5–3.0 μg/L for standing water surrounding two crops, while Main et al. (2014) measured mean and maximum concentrations of 0.14 and 3.1 μg/L, respectively, in water bodies beside canola fields. Measurements for fipronil in the aquatic environment have been reported at 0.17 μg/L (Stark and Vargas 2005); a median of 0.23 and range of 0.004–6.4 μg/L (Mize et al. 2008); 1 μg/L (Hayasaka et al. 2012); and 0.15–5 μg/L (Wirth et al. 2004).

Imidacloprid LC_50_ measurements for fish and amphibia (Table 1) range from 1,200 to 366,000 μg/L, and for clothianidin, from 94,000 to 117,000 μg/L (fish only). Thus, except in the most extreme cases, environmental concentrations are from approximately 2 to 7 orders of magnitude lower than LC_50_ measurements for fish and amphibians, so it is unlikely that the mortality rates of these taxa will be directly affected by these two insecticides under normal exposure. However, the possibility of sub-lethal effects, e.g. physiological stress and damage to DNA, cannot be ruled out (Table 2). For fipronil, there is a greater apparent risk to fish survival, as some of the highest environmental concentrations are within an order of magnitude of their LC_50_ values (Table 1), especially for bluegill sunfish and Nile tilapia. Sub-organism effects may also be apparent, for example, erythrocyte damage and alterations to gene transcription (Table 2).

#### Risks to terrestrial vertebrates

Determining the exposure risks to terrestrial vertebrates is more complex than to aquatic species given that there are several routes of exposure, e.g. from ingestion of treated seed; from residues in or on the crop and soil; from drinking water, nearby vegetation or invertebrates; from dermal exposure due to direct overspray or contact with treated surfaces; from inhalation; and even from preening. Concentrations to which terrestrial taxa can be exposed vary markedly within and between these different pathways, based on habitat requirements and movement between contaminated and uncontaminated patches.

Treated seeds contain some of the highest concentrations of neonicotinoids, with a typical individual canola (oilseed rape), beet or corn seed calculated to contain 0.17, 0.9 or 1 mg of active ingredient, respectively (Goulson 2013). Application rates vary widely by crop but, for example, canola seeds treated with clothianidin have recommended application rates of 4.0 g a.i./kg of canola seed, while corn is almost double, at 7.5 g a.i./kg seed. Given these high concentrations, and that many granivorous species eat crop seeds, the most likely route of exposure to terrestrial animals is probably through the consumption of treated seeds.

Residues in crops and surrounding soil may be lower but still pose a risk to wildlife consumers that feed on the treated plants or ingest soil. For example, Bonmatin et al. (2005) found residues of 2.1–6.6 μg/kg of imidacloprid in seed-treated maize plants. Substantially higher concentrations of 1.0–12.4 mg/kg of imidacloprid have been detected in seed-treated sugar beet leaves (Rouchaud et al. 1994). Ground-dwelling species may also be exposed via the soil. Anon (cited in Goulson 2013) found concentrations of 18–60 μg/kg of imidacloprid in soil following several years of repeated applications as a seed treatment on winter wheat. Donnarumma et al. (2011) measured concentrations of 652 μg/kg of imidacloprid in soil 30 days after sowing of dressed maize seeds, falling to 11 μg/kg at harvest. Following soil drenching (i.e. applying a diluted insecticide directly to the base of a plant), Cowles et al. (2006) found concentrations of 120–220 μg/kg of imidacloprid in hemlock, *Tsuga Canadensis*, tissue. Cutler and Scott-Dupree (2007) found residues of 0.5–2.6 μg/kg of clothianidin in seed-treated canola plants, while Krupke et al. (2012) found residues of 1–9 μg/kg of clothianidin on natural vegetation surrounding seed-treated maize fields. Krupke et al. (2012) also detected concentrations of 6.3 μg/kg of clothianidin in soil in fields sown with seed-treated maize.

The US EPA modelled the estimated daily intake of clothianidin, assuming that mammals and birds only eat a diet of treated seeds (DeCant and Barrett 2010). This risk modelling approach showed that clothianidin, at least when used to treat oilseed rape and cotton seeds, could reduce the survival of small birds and mammals (DeCant and Barrett 2010).

Similar approaches have been developed for other routes of exposure beyond ingestion of seed treatments (e.g. SERA 2005; US EPA 2012). For example, risk modelling for imidacloprid suggests hazards to birds and mammals consuming vegetation, grass and even insects. In particular, it predicts that foliar spraying may lead to substantial mortality of sensitive bird species (SERA 2005). In its 2008 re-assessment of imidacloprid, the US EPA (2008) reported an incident where grubs surfacing after a lawn treatment appear to have poisoned young robins, *Turdus migratorius*.

A more detailed assessment of the risk of intoxication of birds following the consumption of neonicotinoid-treated seed is given by Mineau and Palmer (2013). Their analysis suggests that the risks of acute intoxication with imidacloprid applied on maize, oilseeds or cereals are comparably high, such that birds need only to ingest a few treated seeds. The risk of acute intoxication with clothianidin in maize is highest, whereas for oilseeds or cereals, birds would need to ingest more, largely because application rates are lower. In principle, there are ways in which this risk could be mitigated, for example, by burying seeds below the soil surface, but this is rarely 100 % effective due to spillage (de Leeuw et al. 1995; Pascual et al. 1999). Whether or not birds avoid eating treated seeds (Avery et al. 1998), or the extent to which they may remove a substantial proportion of the toxicant by discarding outer seed husks (Avery et al. 1997) have been debated. However, incidents of bird poisoning with imidacloprid-treated seed have been documented (Berny et al. 1999), suggesting that the calculated risk may be real.

The potential risk to birds from eating neonicotinoid-treated seeds can be illustrated by the following example in which we calculate the relative risk for two granivorous species, a grey partridge, *Perdix perdix* (mass ~390 g) and a house sparrow (mass ~34 g) (http://blx1.bto.org/birdfacts/results/bob3670.htm), feeding on a field recently sown with imidacloprid-treated beet seed, each containing 0.9 mg of imidacloprid (Anon 2012). Imidacloprid is highly toxic to both species, with a LD_50_ of 13.9 mg/kg of body weight for grey partridge and 41 mg/kg for house sparrow (Table 1). Consequently, ingestion of just 6 and 1.5 seeds would have a 50 % chance of killing an individual foraging partridge and sparrow, respectively. Less than a quarter of a seed could have a sub-lethal effect on a house sparrow, as 6 mg/kg is sufficient to reduce flying ability (Table 2; Cox 2001). While de Leeuw et al. (1995) suggest that only 0.17 % of beet seeds remain on the soil surface after sowing, at a maximum drilling rate of 130,000 seeds per hectare (Anon 2012), 6 and 1.5 seeds would be found on the surface in areas of approximately 270 and 70 m^2^, respectively, well within the daily foraging ranges of each species. Areas of accidentally spilled seed could contain much higher densities. While individual partridges and sparrows may not ingest treated seeds (i.e. as the brightly coloured seed coatings may deter birds if they represent a novel food source), these calculations suggest that there is a potential risk of imidacloprid-treated seeds to affect sensitive bird species, consistent with conclusions drawn by DeCant and Barrett (2010), Mineau and Palmer (2013) and Goulson (2013). Anecdotal observations of blackbirds and sparrows foraging in fields recently seeded with neonicotinoid-treated crops suggest that the calculated risks are further plausible (C. Morrissey personal observation).

## The indirect effects of pesticides on vertebrate wildlife

While rarely considered in ecological risk assessments, concerns about the impacts of pesticide use on vertebrates have more recently turned to the widespread potential for indirect effects (Sotherton and Holland 2002; Boatman et al. 2004). Observations of farmland and grassland bird declines and range contractions correlate well with agricultural intensification, including increased pesticide use (Chamberlain et al. 2000; Morris et al. 2005; Ghilain and Bélisle 2008; Robillard et al. 2013; Mineau and Whiteside 2013). Tennekes (2010) and Mason et al. (2012) have recently suggested, albeit with little supporting evidence, that neonicotinoid insecticides may be contributing to declines of insectivorous birds in Europe, and of fish, amphibians, bats and birds around the world, respectively. Tennekes (2010) hypothesized that neonicotinoids were acting indirectly on bird populations, by reducing the abundance of their insect prey. Mason et al. (2012) suggested that neonicotinoids have suppressed the immune system of vertebrates (and invertebrates) making them more prone to infectious disease and other stressors.

Indirect effects of pesticides on vertebrates are most commonly exerted in one of three ways: (1) through reductions of plant seed food for granivores following herbicide applications (e.g. Gibbons et al. 2006); (2) through the loss of insect host plants following herbicide applications and the secondary impacts for dependent insects and insectivores, (e.g. Potts 1986); or (3) through reductions in arthropod prey for insectivores following applications of insecticides—or fungicides with insecticidal properties (e.g. Martin et al. 2000; Morris et al. 2005; Poulin et al. 2010).

Indirect effects are inherently difficult to measure and frequently suffer from limitations of correlative inferences. Boatman et al. (2004) highlighted three criteria for conclusively inferring a causal link between pesticides and their indirect actions on vertebrate wildlife. Conclusive studies should document negative effects on (1) food quality and quantity, (2) reproduction, condition or survivorship of the vertebrate consumer and (3) concomitant vertebrate population declines. The only documented case where indirect effects were definitively shown using the full range of these criteria in a fully replicated field experiment was for the grey partridge in Britain (Rands 1985) following several decades of intensive study. Population modelling showed that declines in grey partridge populations could be wholly explained by herbicide-induced reductions in prey availability in tandem with reduced growth and survival of grey partridge chicks (reviewed by Potts 1986). Other studies, however, have revealed consistent effects on one or more of these three criteria, suggesting that the indirect effects of pesticides may be more prevalent than documented in the literature.

### Studies reporting effects on consumers through food reductions

Pesticide applications, in temperate regions, directly overlap with the seasonal production of invertebrates and the breeding seasons of a range of numerous vertebrate species. Food supply (i.e. abundance and availability) is widely accepted as affecting habitat selection, reproductive success and survival in vertebrates, with extensive supporting evidence for birds in particular (Simons and Martin 1990; Johansson and Blomqvist 1996; Brickle et al. 2000; Moller 2001; Hole et al. 2002; Nagy and Holmes 2004, 2005; Boatman et al. 2004; Morris et al. 2005; Britschgi et al. 2006; Hart et al. 2006; Zanette et al. 2006; Golawski and Meissner 2008; Selås et al. 2008; Dunn et al. 2010; Poulin et al. 2010). Across Europe and North America, dramatic and widespread declines have been observed in populations of birds associated with farmland and wetland habitats (Beauchamp et al. 1996; Donald et al. 2001; Benton et al. 2002; Boatman et al. 2004), with arthropod abundance showing similar trends (Benton et al. 2002). In Canada and the USA, however, species loss has been more strongly correlated with pesticide use than agricultural area or intensification measures alone (Gibbs et al. 2009; Mineau and Whiteside 2013).

Reductions in invertebrate food abundance caused by insecticide use has been linked to reductions in reproductive success of at least four farmland passerines in the UK: corn bunting, *Miliaria calandra*, yellowhammer, *Emberiza citrinella*, whinchat, *Saxicola rubetra*, and reed bunting, *Emberiza schoeniclus* (Brickle et al. 2000; Brickle and Peach 2004; Morris et al. 2005; Hart et al. 2006; Dunn et al. 2010; but see Bradbury et al. 2000, 2003). Although declines in bird populations in the UK have been coincident with invertebrate losses, changes in invertebrate abundance alone do not fully explain population trends for these species. In fact, the nesting success of these species increased during time periods when populations were declining (Siriwardena et al. 2000). Population declines of seed eaters have instead been linked to reduced over-winter survival, likely as a consequence of reduced seed availability (Siriwardena et al. 2000; Butler et al. 2010).

### Indirect effects of neonicotinoids and fipronil

We found only six studies that have investigated the indirect effects of neonicotinoids and fipronil on vertebrate wildlife (Table 3). All were field rather than laboratory-based studies. Of these studies, one found a beneficial, indirect effect. Female Cape ground squirrels, *Xerus inauris*, benefited from ectoparasite removal with fipronil and had fourfold higher breeding success (Hillegass et al. 2010). A number of studies have shown that reducing parasite burdens can enhance vertebrate breeding success (e.g. Hudson et al. 1992). However, interpretation of the effect of fipronil was not straightforward, as endoparasites were simultaneously removed with ivermectin, and researchers could not distinguish the effects of the two products.

**Table 3:** Indirect effects of imidacloprid and fipronil on vertebrates

Taxon and Species	Effect on:	Imidacloprid	Fipronil	Source and detailed effect
Mammal				
Lesser hedgehog tenrec, *Echinops telfairi*	Population		REC	Peveling et al. 2003; marked reduction in harvester termite prey may eventually lead to tenrec decline
Cape ground squirrel, *Xerus inauris*	Reproduction		0.7 mg/kg; REC (POS)	Hillegass et al. 2010; removal of ectoparasites (with fipronil) and endoparasites boosted breeding success; unable to determine impact of fipronil alone
Bird				
3 neotropical migrant insectivores	Population	REC (NE)		Falcone and DeWald 2010; spraying reduced lepidopteran prey, but not populations of black-throated green warbler (*Dendroica virens*), black-throated blue warbler (*D. caerulescens*) and blue-headed vireo (*Vireo solitarius*)
38 species, of which 33 were insectivores	Population		REC (NE)	Norelius and Lockwood 1999; marked reduction in grasshoppers, but not in bird densities; 34 bird species studied, most abundant were horned lark, *Eremophila alpestris*, western meadowlark, *Sturnella neglecta*, and lark sparrow, *Chondestes grammacus*
Fish				
Medaka, *Oryzias latipes*	Growth & development	0.001 mg/L; REC	0.001–0.05 mg/L; REC	Hayasaka et al. 2012; reduced growth of both adults and fry
Japanese carp, *Cyprinus carpus*	Growth and survival		REC (NE)	Clasen et al. 2012; no effect on growth and survival of Japanese carp
Reptile				
Madagascar iguana, *Chalarodon madagascariensis*	Population		REC^7^	Peveling et al. 2003; marked reduction in harvester termite prey led to decline in iguana population
A skink, *Mabuy elegans*	Population		REC^7^	Peveling et al. 2003; marked reduction in harvester termite prey led to decline in skink population

All other studies demonstrated deleterious effects

*REC* insecticide applied at manufacturer’s recommended rate, *NE* no effect at the given dosage, *POS* positive effect at the given dosage

In two further field studies, both in experimental rice fields, imidacloprid and/or fipronil was applied at the recommended commercial rates. While one study found no effect of fipronil on growth or survival of Japanese carp, *Cyprinus carpio* (Clasen et al. 2012), the other found that both imidacloprid and fipronil applications reduced the growth of both adult and fry medaka fish, *Oryzias latipes* (Hayasaka et al. 2012). Hayasaka et al. (2012) suggest that this is most likely an indirect effect, through a reduction in the abundance of medaka prey. The concentrations were probably too low (approximately 0.001 to 0.05 mg/L) to exert a direct toxic effect on medaka but assumed sufficiently high to reduce the abundance of their invertebrate prey.

Population-level studies investigating indirect impacts of neonicotinoids and fipronil on vertebrate species are rare. Only three such studies were found during this review, and all were of local—rather than national or regional—populations (Table 3). All were field studies that applied either imidacloprid or fipronil at recommended commercial rates using sprays or soil drenching, rather than seed treatments.

Falcone and DeWald (2010) investigated the impact of a single soil drenching application with imidacloprid on eastern hemlock, *Tsuga Canadensis*, as part of a campaign to reduce numbers of an exotic insect pest. While the soil drenching had (surprisingly) no impact on the woolly adelgid (*Adelges tsugae*) pest, populations of non-target hemiptera and lepidoptera were reduced. Despite lepidopteran larvae being important in the diet of three neotropical migrant insectivorous bird species, bird numbers were not affected in the following year. Norelius and Lockwood (1999) undertook a similar study, this time spraying with fipronil to control a grasshopper outbreak. While grasshopper numbers were markedly reduced, populations of insectivorous prairie birds that commonly consume the grasshoppers were slightly, but not significantly, reduced a month after spraying. The lack of clear population-level effects in both these studies may have been related to birds seeking food outside treated areas in compensation, although this seems unlikely, at least for the Norelius and Lockwood (1999) study, as the home ranges of the birds studied (few hectares) were small compared to the total treated area (few hundred hectares). Alternatively, population-level effects could have been masked in such relatively small-scale field trials if birds had immigrated into the treated plots from surrounding un-treated areas. Neither study, however, measured breeding success or impacts on chick survival which may be more plausible than effects on adult survival.

In contrast, Peveling et al. (2003) documented how fipronil spraying to control a plague of migratory locusts in Madagascar halved populations of the harvester termite, *Coarctotermes clepsydra*. Consequently, populations of two lizard species, the Madagascar iguana, *Chalarodon madagascariensis*, and a skink, *Mabuy elegans*, declined, because termites form an important part of the diet of both species, while the lesser hedgehog tenrec, *Echinops telfairi*, may have also been affected. To date, this is the only study that has demonstrated a population-level impact of a systemic insecticide on a vertebrate population, where its effect was exerted indirectly through the food chain. While Tingle et al. (2003) report that a study of fipronil spraying to control locusts in Madagascar may have caused population declines of two bird species, Madagascar bee-eater, *Merops superciliosus*, and Madagascar kestrel, *Falco newtoni*, (but no effect on two others, Madagascar bush lark, *Mirafra hova*, and Madagascar cisticola, *Cisticola cherina*), sample sizes were too small to be conclusive, and it was not possible to distinguish between direct and indirect effects.

While it is possible to use laboratory toxicity studies to inform models on the indirect effects of a pesticide on vertebrate populations, such models are very data-demanding and case studies are rare (see e.g. Watkinson et al. 2000). Systemic insecticides are known to affect invertebrate populations (e.g. Whitehorn et al. 2012; Van Dijk et al. 2013), but the lack of evidence for, and difficulty in determining, comparable indirect effects on vertebrates is an issue in ecotoxicology. There remains an essential need to determine if a causal link between loss of insect prey through pesticide use and the decline of vertebrate populations exists. This is especially true in North America and Europe where neonicotinoids are being used in large quantities and over vast areas.

## Conclusions

Neonicotinoid and fipronil insecticides can exert their impact on vertebrates either directly, through their overt toxicity, or indirectly, for example, by reducing their food supply. Marked variation exists among taxa and different systemic insecticides in acute toxicity (as measured by LD_50_ and LC_50_), while a range of sub-lethal effects can occur at concentrations orders of magnitude below those causing lethality. Overall, at concentrations relevant to field exposure scenarios from seed treatments (birds) or water concentrations (fish), imidacloprid and clothianidin can be considered a risk to granivorous bird species, while fipronil may pose a similar risk to sensitive fish species. Except in the most extreme cases, however, concentrations of imidacloprid and clothianidin that fish and amphibians are exposed to appear to be substantially below thresholds to cause mortality, although sub-lethal effects have not been widely studied.

Despite the lack of research and the difficulty in assigning causation, indirect effects may be as—or even more—important than direct toxic effects on vertebrates, as modern systemic insecticides are more effective at killing the invertebrate prey of vertebrates than the vertebrates themselves. Given the data here, current risk assessment procedures for neonicotinoids and other systemic pesticides need to consider the associated risks from both direct and indirect effects to vertebrate wildlife.
